# Laser-Directed Energy-Deposited Ti-6Al-4V: The Anisotropy of Its Microstructure, Mechanical Properties, and Fracture Behavior

**DOI:** 10.3390/ma18102360

**Published:** 2025-05-19

**Authors:** Huan Wang, Chen-Wei Liu, Tianyu Wu, Hua-Xin Peng

**Affiliations:** Institute for Composites Science Innovation (InCSI), School of Materials Science and Engineering, Zhejiang University, Hangzhou 300027, China; hwang2014@zju.edu.cn (H.W.); 22126042@zju.edu.cn (C.-W.L.); 22260429@zju.edu.cn (T.W.)

**Keywords:** additive manufacturing (AM), laser-directed energy deposition (L-DED), Ti-6Al-4V (Ti64), mechanical properties anisotropy, grain boundary α

## Abstract

Ti-6Al-4V (Ti64) is widely used in the additive manufacturing (AM) industry for its superior mechanical properties; however, severe anisotropy is inevitable. In this work, a Ti64 sample fabricated using laser-directed energy deposition is used for fundamental investigations into the anisotropy of its microstructure, mechanical properties, and fracture behaviors. The microstructure of martensite α and prior β-Ti grains are characterized in both the XOY and XOZ planes. The tensile/compressive properties and microhardness along the building direction (BD) and scanning direction (SD) are tested, and it is found that the sample along the SD has better comprehensive mechanical properties. Due to grain boundary α (GB-α), different fracture behaviors and crack propagation paths are found along the BD and SD. When tensile force is parallel to the growth orientation of GB-α, a much higher density of microcracks caused by fractured GB-α is found to contribute to a prolonged elongation and the weakening of strength. While stretching along the SD, the cracks would propagate along the GB-α easily and straightly, which might lead to lower elongation.

## 1. Introduction

In the past few decades, the titanium alloys have been attracting growing research interest [1,2]. Ti-6Al-4V (Ti64), one of the most promising (α + β) type titanium alloys, has merits of high specific strength/stiffness [3], preeminent corrosion [4], and heat resistance [5], for which it is widely employed in various fields, i.e., aerospace [6], vehicle, and biomedical fields [7]. Nevertheless, with the increasing demand for complicated components and structures, including hollow [8], inner, and thin-walled structures [9], the traditional fabrication methods for Ti64 [10] appear to be insufficient due to high cost and low flexibility. Thus, the novel fabrication method for Ti64 with complicated architectures is required.

Laser-directed energy deposition (L-DED) is one of the most attractive metal/alloy additive manufacturing (AM) methods for its features of material saving and fast and near-net shaping ability and has been widely utilized in aerospace and component fabrication industries [1]. During the deposition, the metal powders spurt out from the feeding nozzles and are rapidly melted into a molten pool with a laser beam employed as an energy source [11]. The molten pool solidifies with an extremely high cooling rate of 10^3^–10^4^ K/s to form components layer by layer as programmed [12]. The Ti64 alloy is one of the most suitable materials for the L-DED technique due to its superior properties and formability [13]. Moreover, the fabrication process of printing layer by layer also contributes to the higher design flexibility and potential in weight reduction [14].

Though with advantages and superiority, the problems, including residual stress, uncontrollable defects, microstructure, and properties anisotropy, are inevitable and greatly constrain the development of Ti64 via AM [15]. Hot isostatic pressing and field-assisted metal additive manufacturing methods are proposed as the solutions with which these problems could be relieved to some degree; however, they are still not avoidable [16]. With a higher temperature gradient along the Z direction (building direction, BD), Ti64 commonly exhibits equiaxed prior β-Ti grains along the SD (scanning direction), while column-like crystals span several deposited layers along the BD [17]. Such a microstructure always leads to property anisotropy, whether for mechanical [18,19] or chemical properties [20]. Meanwhile, it is reported that the phenomenon of grain boundary α (GB-α) [21] (also known as the α layer [22]), the oversized α grains along the prior β-Ti grain boundaries, could cause a reduced elongation/toughness and enhanced property anisotropy [23]. However, how GB-α behaves during the tensile process and how it contributes to mechanical property anisotropy and affects microcrack propagation along different directions must be further discussed.

For further understanding of the anisotropy of the microstructure, mechanical properties, and fracture behavior of Ti64 via L-DED, here, in our work, the Ti64 samples were fabricated using the L-DED AM technique with optimized printing parameters. The microstructures in the XOY and XOZ planes were characterized, compared, and analyzed. The mechanical properties (tensile/compressive properties and hardness) along the BD and SD were tested and compared. Moreover, to reveal the anisotropy of fracture behaviors along both directions, the work also emphasizes how GB-α contributes to different microcrack propagation paths while stretched along both directions. This work accumulates experience for Ti64 alloy using the L-DED technique, making further understanding into the mechanical property anisotropy and the role that the GB-α phenomenon plays in sample fracture.

## 2. Materials and Methods

### 2.1. Materials and Powders Treatment

**Figure 1:**
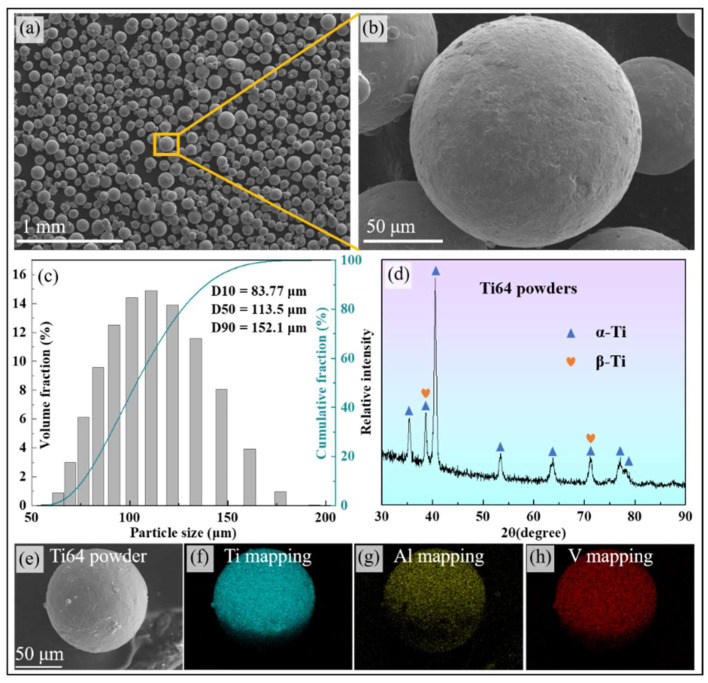
Ti64 powder characterization. (**a**) SEM image of Ti64 powders used for L-DED. (**b**) Magnified powder framed in (**a**) by yellow lines. (**c**) Diameter distribution and (**d**) XRD spectrum of Ti64 powders used for L-DED. (**e**–**h**) Element distribution (Ti, Al, and V) maps of Ti64 powder.

In this work, the Ti64 powders (Zhongyuan Co., Ltd., Ningbo, China) with spherical shapes were used for sample fabrication (Figure 1a,b). The Ti64 powders utilized had a diameter distribution of D10 = 83.8 μm, D50 = 113.5 μm and D90 = 152.1 μm (Figure 1c). The particle size is estimated and calculated via laser particle size analyzer (Beckman Coulter, LS 13320, Suzhou, China). The X-ray diffraction (XRD) spectrum suggested that the powders were composed of both α-Ti and β-Ti phases, and no diffraction peaks of TiO_2_ were detected (Figure 1d). The powders were examined using energy dispersive spectroscopy (EDS), and the elements (Ti, Al, and V) were distributed homogeneously without aggregation (Figure 1e–h). The Ti64 powders were all vacuum dried for at least 1 h at 120 °C.

### 2.2. Sample Fabrication and L-DED Process

**Figure 2:**
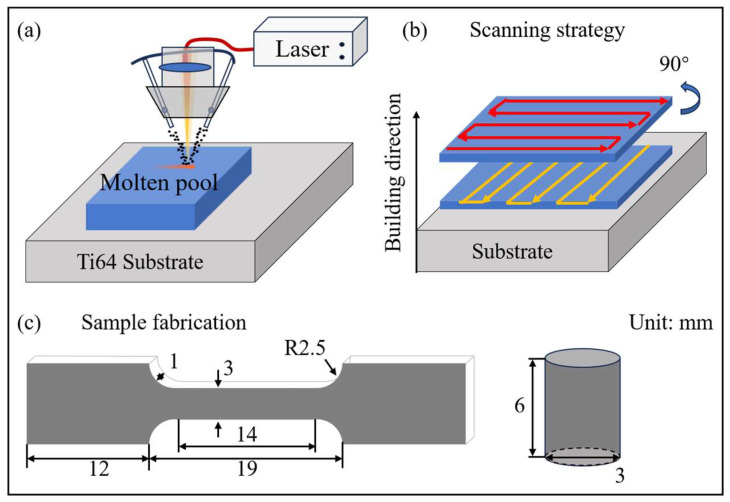
Schematic diagrams of sample fabrication. The schematic illustrations of (**a**) the L-DED process, (**b**) the scanning strategy, and (**c**) the tensile and compressive samples, respectively.

The Ti64 powders were vacuum dried at 120 °C for at least 1 h before they were applied to L-DED (BLT-C400, Xi’an Bright Laser Technologies Co., Ltd., Xi’an, China) for a higher printing quality. During the L-DED printing process, Ti64 powders were placed into a powder feeder and then fed into a molten pool using four-way coaxial powder feeding nozzles with the pressure of an argon atmosphere. The powders were instantaneously melted into the molten pool by a laser beam and then solidified with an extremely high cooling rate. The whole deposition process and the sample were protected from sample oxidation by a 99.999% argon shield atmosphere (Figure 2a). The scanning strategy used was such that (Figure 2b) the laser scanning direction rotated 90° between adjacent layers to release thermal stress and weaken anisotropy [24]. The samples for the tensile test were wire-cut, in a dog-bone-like shape with a 14 mm-long parallel length. The compressive samples had dimensions of Φ3 h6 (Figure 2c). The tested samples were all prepared in both the building direction (BD, XOZ plane along the Z axis) and scanning direction (SD, XOZ plane along the X axis).

**Figure 3:**
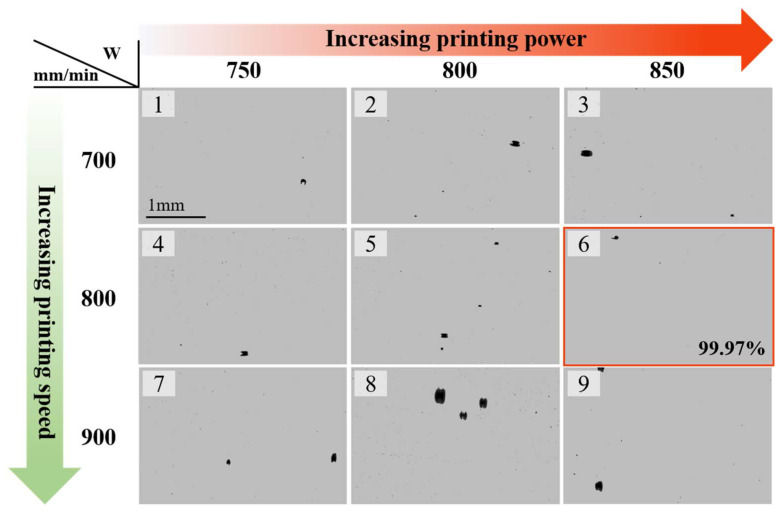
Optical microscope images of polished as-printed Ti64 samples with varying printing parameters. The optimized parameters are framed (red line) with a 99.97% relative density. The relative density is calculated using the area fraction of non-pore regions.

For the optimized printing parameters, including laser power, scanning speed, and layer thickness, the orthogonal pre-experiments were conducted. The single printing path experiment was applied to confirm the layer thickness with varying energy density, which is shown in Figure S1 in the Supplementary Materials. The layer thickness was then utilized for corresponding cubic sample fabrication with varying parameters [25]. The samples were polished for the relative density calculation (Figure 3), which was measured using the non-pore region area fraction [26]. The printing parameters were confirmed using the index of maximum relative density, which was calculated using the commercial MATLAB software (MATLAB 2016A). With the optimized printing parameters (a laser power of 800 W, a scanning speed of 800 mm/min, a layer thickness of 0.4 mm, a printing pressure of 0.6 MPa, an overlapping rate of 50%, and an oxygen level under 200 ppm with a feeding rate of 2.5 g per minute), the relative density was 99.97% (framed with red lines in Figure 3).

### 2.3. Microstructure Characterization and Mechanical Properties Tests

In this work, the polished samples and optical microscope (OM, MSD4300, Murzider, Beijing, China) were utilized for relative density and defect calculation. The commercial MATLAB software was used for image processing and relative density confirmation. For each sample, nine positions were observed in total for average relative density. The samples were polished and then etched using a Kroll reagent (HF:HNO_3_:H_2_O = 5:15:80 vol.%) for 15 s before being applied to the scanning electron microscope (SEM, Gemini 300, ZEISS, Oberkochen, Germany) for observation. Energy dispersive spectrometer (EDS) mapping was employed for element distribution characterization for the Ti64 powders. The sample was mechanically polished and then argon ion polished (AIP, EM TIC3X Leica, Wetzlar, Germany) before being utilized for electron backscatter diffraction (EBSD, symmetry2, Oxford, British) with a scanning step of 0.1 μm for grain morphology characterization. The EBSD results were analyzed using Aztec software, following the ISO 13067 [27] international standard. The X-ray diffraction (XRD, XRD-6000, SHIMADZU, Tokyo, Japan) was conducted for Ti64 powder phase identification, scanning from 30° to 90° with a scanning speed of 3° per minute. The XRD data were analyzed using Jade software.

A universal testing machine (CMT-5205, Sans, Shenzhen, China) with a 10 mm/6 mm extensometer was used for quasi-static uniaxial tensile and compressive tests, respectively. The tests were operated with a strain rate of 10^−3^/s. An HV testing machine (Duramin-40, Struers, Shenzhen, China) was used for microhardness tests with a 10 s-long 2 N load, and 36 points in total within a 1 mm × 1 mm square area were counted for the average hardness.

## 3. Results and Discussion

### 3.1. Microstructure Anisotropy

**Figure 4:**
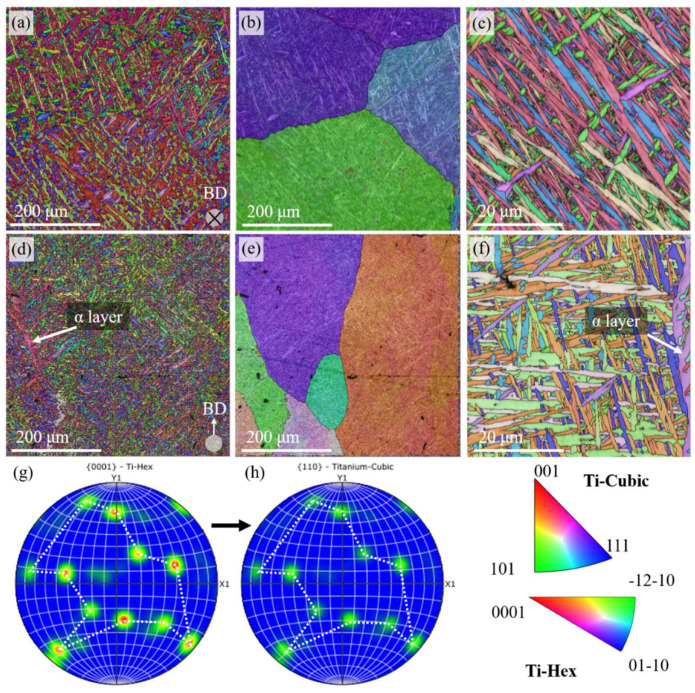
EBSD results of the Ti64 sample. Large-scope inversed pole figure (IPF) images of the Ti64 sample in the (**a**) XOY and (**d**) XOZ planes. Parent grain reconstruction results in the (**b**) XOY and (**e**) XOZ planes. High-magnification IPF images in the (**c**) XOY and (**f**) XOZ planes. (**g**) The grain orientation pole figure of the α-Ti grains in (**d**) and (**h**) the grain orientation pole figure of the β-Ti grains in (**e**).

EBSD is utilized for microstructure and grain morphology characterization. The large-scale EBSD results are shown (Figure 4a,d), and the α’ grains are found in lath-like shapes, which are caused by the martensitic transformation from β-Ti grains to α’-Ti grains with an extreme cooling rate [28]. With the parent phase reconstruction technique, the prior β-Ti grains are firmed (Figure 4b), which are in equiaxed grains with a grain size of nearly 500 μm (calculated by the diameter of the equivalent circle). EBSD in a relatively small scope with high resolution is used for α’-Ti grain size calculation (Figure 4c). The α’-Ti grains in the XOY plane have a grain size of 0.88 μm. Generally, the grain morphologies of α’-Ti grains in samples fabricated using other AM methods, laser-powder bed fusion (L-PBF) for example, are nearly the same, while the grain sizes are finer than those fabricated using L-DED, for L-PBF is characterized by a much higher cooling rate than that of L-DED [29].

For example, in the XOZ plane, some oversized grains in irregular shapes are commonly observed (arrowed in Figure 4d). The oversized α’-Ti grains distributed along the prior β-Ti grains (which can be confirmed with the result of parent grain reconstruction in Figure 4e) are called the α layer or grain boundary α (GB-α), which are believed to be detrimental to toughness and provide a preferred orientation for crack propagation during tensile tests [23]. In addition, the prior β-Ti grains are with a column-like shape, spanning several printing paths, which is caused by the much higher temperature gradient along the Z axis than others (Figure 4e). Such morphology, an α layer along the column-like prior β-Ti grains, would definitely contribute to the anisotropy of microcrack propagation behaviors along the BD and SD. At the same time, the average size of α’-Ti grains in the XOZ plane is 1.10 μm, which is a little larger than that in the XOY plane (Figure 4f).

For the grain orientation relationship exploration, the pole figures (Figure 4g,h) corresponding to Figure 4d and e are shown. A slight texture is observed. Moreover, the grain orientation of both α and β-Ti grains highlighted in the figure is coincident, proving the Burgers relationships [30] during martensitic transformation. The same coincident pore figures are also found in the XOY plane (Figure S2 in the Supplementary Materials).

### 3.2. Mechanical Properties Anisotropy

**Figure 5:**
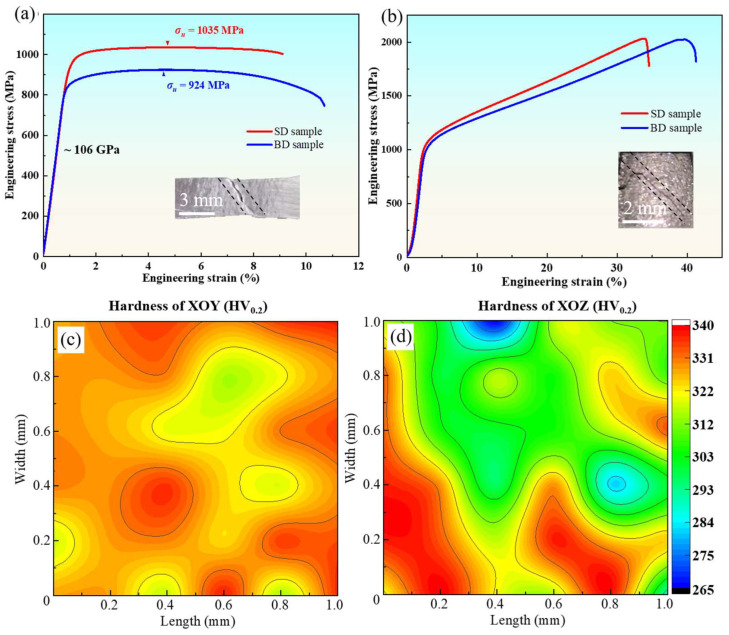
Mechanical properties of Ti64 samples. Typical engineering strain–stress curves during (**a**) tensile and (**b**) compressive tests of the Ti64 samples along both the BD and SD. The main cracks of fractured samples are shown in the inset. Microhardness distribution maps of the as-printed Ti64 sample in (**c**) the XOY plane and (**d**) the XOZ plane. The average hardness is calculated using 36 points within one square millimeter.

The mechanical properties, including tensile and compressive properties and microhardness along the BD and SD, are tested and shown (Figure 5) to reveal the property anisotropy. For the tensile test, the Ti64 samples in the BD and SD perform with nearly the same Young’s modulus (around 106 GPa) and uniform elongation (4.7% and 4.6% for SD and BD samples, respectively). The SD sample exhibits an obviously higher ultimate tensile strength (1035 MPa, 12% higher than the BD sample) than that of the BD sample (924 MPa), which might be related to the microstructure distinguishment. Moreover, the BD samples possess a significant softening stage and a non-devastating fracture with a prolonged elongation, indicating a more obvious necking phenomenon than that in the SD (Figure 5a). For compressive properties, the same stiffness is also found for samples in both directions. Though the work hardening rate of the SD samples is stronger, the ultimate compressive strength is similar for the BD and SD samples. The compressive strain is around 34% and 40% for the SD and BD samples, respectively (Figure 5b). In the inset the figure, it could be found that, whether for tensile or compressive tests, the main cracks propagate along roughly 45°, suggesting ductile damage with maximum shear stress [31]. It is noted that Ti64 samples fabricated via L-PBF with optimized printing parameters always perform with higher strength, which can be around 1200 MPa [29]. The higher strength is caused by finer microstructures/α-Ti grains and fewer defects during fabrication; however, longer manufacturing time is the cost.

The microhardness of both planes was tested, and the XOY plane (328.42 Hv, Figure 5c) was found to have a slightly higher average hardness than the XOZ plane (316.40 Hv, Figure 5d). Moreover, the standard deviation of hardness in the XOY plane was 6.5 Hv, while that was obviously larger in the XOZ plane (11.3 Hv), which might be caused by the microstructure nonuniformity and column-like prior β grains. In short, the sample in the SD performs better in terms of comprehensive mechanical properties, and the anisotropy is inevitable for the as-printed Ti64 sample; however, the difference for the BD and SD is within an acceptable range (around 10%).

### 3.3. Fracture Behaviors and Crack Propagation During Tensile Tests

**Figure 6:**
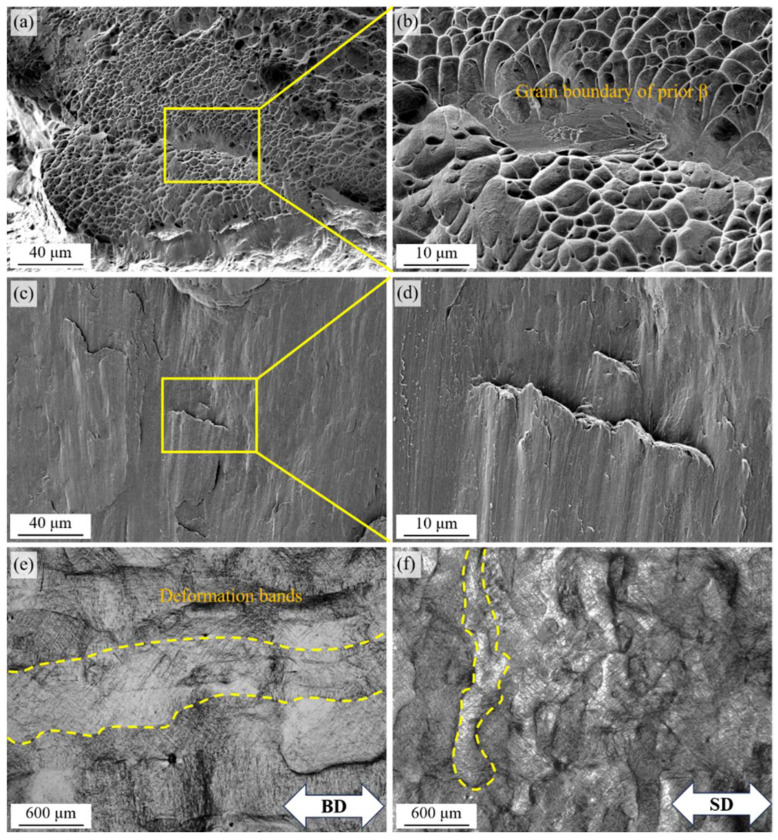
Fracture surfaces of Ti64 samples. The fracture surfaces of (**a**) the tensile sample and (**b**) the magnified region framed with yellow lines. The fracture surfaces of (**c**) the compressive sample and (**d**) the magnified region framed with yellow lines. The profiles of the fracture samples in (**e**) the BD and (**f**) the SD; the deformation bands are framed with yellow dashed lines.

The fractured surfaces of tensile and compressive samples are shown (Figure 6a–d), and no significant differences were found between the samples in the BD and SD. Dimples were observed as the dominant morphology in the tensile fracture (Figure 6a), and the dimples were nearly the same size and depth, indicating good toughness and a uniform microstructure. In the magnified the region framed with yellow lines, a lath crack with a different morphology was found (Figure 6b), which was caused by a crack along the GB-α. GB-α always possesses worse ductility, and microcracks would form and propagate along it [32]. On the surface of the compressive fracture, no dimples were found (Figure 6c,d), suggesting that the compressive fracture was caused by microcrack aggregation.

Besides the fractured surfaces, deformation bands were observed on the surfaces of the samples (Figure 6e,f). Whether the tensile force was along the BD or SD, the deformation bands were all along the prior β-Ti grain boundary, which was caused by the microstructure texture. Within a single prior β-Ti grain, the lath α’ grains had specific grain orientations due to the martensitic transformation, which contributed to a consistent deformation behavior [32]. Thus, the deformation bands around the prior β-Ti grain formed and intensified the non-uniformity of deformation. The same phenomenon was also observed using the in situ SEM method in other works [33]. With tensile load exceeding the yield stress of the sample, the deformation behavior and primary microcracks occurred in prior β-Ti grains along the α’ grains lath. With further strain, more obvious deformation bands were found along the prior β-Ti grain boundaries, which is believed to be detrimental to the uniformity of deformation. The different deformation degrees of adjacent prior β-Ti grains caused by varying grain orientations was proven to be the main reason for the deformation bands [33].

**Figure 7:**
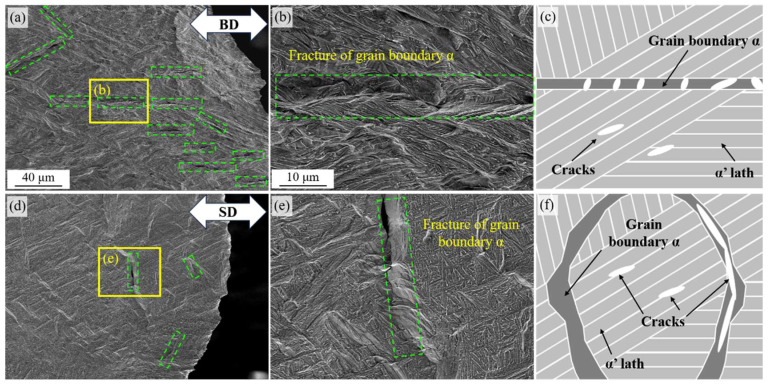
Fractured sample and schematic diagrams of crack propagation. (**a**) Profile of the fractured tensile sample along the BD. (**b**) The magnified image which is framed with yellow lines in (**a**). (**c**) The schematic diagram of crack propagation during tensile tests along the BD. (**d**) The profile of the fractured tensile sample along the SD. (**e**) The magnified image, which is framed with yellow lines in (**d**). (**f**) Schematic diagram of crack propagation during tensile tests along the SD. The microcracks are framed with green dashed lines in the figure.

For further understanding of different crack propagation behaviors along the BD and SD, the etched XOZ planes of the fractured samples are shown (Figure 7). In the sample with tensile force along the BD, numerous microcracks were found and framed with green dashed squares. Moreover, the cracks are nearly all parallel to the stretching direction (Figure 7a). In the magnified region framed with a yellow square, it can be seen that the cracks are along the prior β-Ti grain boundaries, which was caused by the relatively worse toughness and preferred crack nucleation along GB-α. Additionally, the cracks within prior β-Ti grains form near the α lath and propagate along the lath boundary (Figure 7b) due to the stress concentration [34]. It should be noted that when drawn along the BD, the grain boundary α would directly bear the tensile stress, and considerable cracks would prematurely form inside it, which could result in weakened tensile strength. However, fortunately, the cracks will be blunted by the α lath due to better ductility and a different crack propagation direction, which led to a prolonged elongation as tested (Figure 7c).

For the tensile sample along the SD, microcracks along prior β-Ti grain boundaries were also found, which were perpendicular to the tensile direction (framed with green dashed squares in Figure 7d). Nevertheless, it could be found that the density of such microcracks is much lower than that in the BD samples. The magnified crack framed region is marked with yellow lines; the crack was also caused by the fracture of GB-α (Figure 7e). With the tensile load vertical to the GB-α plate, the microcracks expand, propagate, and converge into the main crack more easily along GB-α grain boundaries (Figure 7f), which contributes to lower crack density and reduced elongation. The same assumption and model were also proposed and analyzed by other works [23]. It is hypothesized that the column-like prior-β grain morphology and distribution of GB-α grains enhanced mechanical property anisotropy. When the tensile load is being applied perpendicular to the grain boundaries, the GB-α grains will be subjected to Mode I opening failure. Therefore, with an opening-mode tensile load, the damage and crack propagation would be accelerated, leading to lower ductility and premature fracture [23].

## 4. Conclusions

In this work, a fundamental investigation into the microstructure/property anisotropy of Ti64 via the L-DED technique were tested and analyzed. The different fracture behaviors and crack propagation paths along the BD and SD caused by GB-α were discussed. The following major conclusions can be drawn:

(1) With an extremely high cooling rate, the Ti64 sample exhibited plate-like α’-Ti lath due to martensitic transformation. The prior β-Ti grains were in equiaxed crystals in the XOY plane (with an equivalent diameter of around 500 μm) and in a column-like shape spanning several printing paths in the XOZ plane, caused by the much steeper temperature gradient along the BD. Oversized GB-α with irregular shapes formed along the prior β-Ti grain boundaries.
(2) The sample along the SD had a higher tensile strength (1035 MPa) but a reduced elongation (9.5%) than the sample along the BD (924 MPa, 11.0%). The uniform elongation and compressive properties were similar for both samples. The hardness of the XOY plane (328.42 Hv) was a little higher than that in the XOZ plane (316.40 Hv).
(3) During the tensile test, the surface deformation bands along prior β-Ti grain boundaries could be observed and contributed to the non-uniform deformation. When stretching along the BD, the cracks forming along GB-α had a higher density, but would not easily converge into the main crack, which contributed to a lower strength but prolonged elongation. While stretching along the SD, the cracks forming along GB-α tended to transform into the main crack and led to a reduced elongation.

## Data Availability

The original contributions presented in the study are included in the article, further inquiries can be directed to the corresponding author.

## Supplementary Materials

The following supporting information can be downloaded at https://www.mdpi.com/article/10.3390/ma18102360/s1: Figure S1: The OM images of a single-path experiment of polished as-printed Ti64 samples with varying printing parameters. Figure S2. The pore figures of an as-printed Ti64 sample in the XOY plane.
