# Supplementary material for: Laser-Directed Energy-Deposited Ti-6Al-4V: The Anisotropy of Its Microstructure, Mechanical Properties, and Fracture Behavior
Source: Materials (Basel). 2025 May 19;18(10):2360. doi: 10.3390/ma18102360 (PMC12113225; doi:10.3390/ma18102360)
Supplement: Supplementary file 1 [file materials-18-02360-s001.zip › materials-3604077-supplementary.pdf]

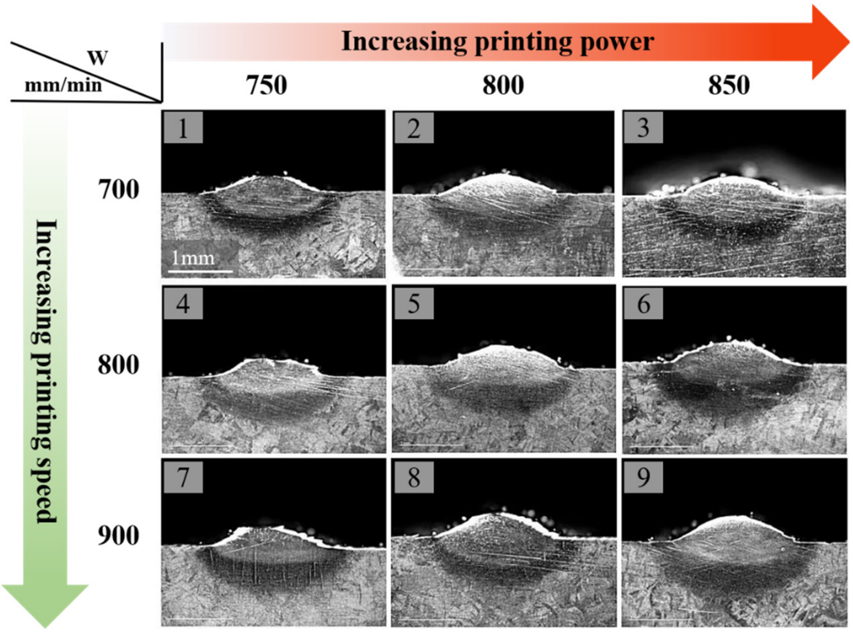

Fig. S1. The OM images of single path experiment of polished as-printed Ti64 samples with varying printing parameters.

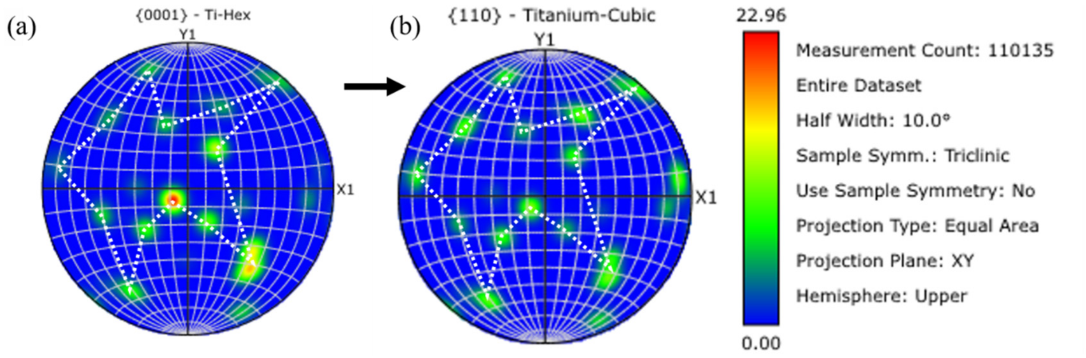

Fig. S2. The pore figures of as-printed Ti64 sample in XOY plane.
